# Self-Supported Polyhedral-like Co_3_S_4_ Nanostructures Enabling Efficient High Current Hydrogen Evolution Reaction

**DOI:** 10.3390/ma18215025

**Published:** 2025-11-04

**Authors:** Abu Talha Aqueel Ahmed, Sangeun Cho, Abu Saad Ansari, Yongcheol Jo, Atanu Jana

**Affiliations:** 1 Division of System Semiconductor, Dongguk University, Seoul 04620, Republic of Korea; talhaphy@gmail.com (A.T.A.A.); sangeun.c@dongguk.edu (S.C.); 2 Nano Center Indonesia Research Institute, Puspiptek Street, South Tangerang 15314, Banten, Indonesia; saad@nano.or.id; 3 Department of Opto-Mechatronics Engineering, Pusan National University, Busan 46241, Republic of Korea; joyc2312@gmail.com

**Keywords:** hydrothermal synthesis, anion-exchange, polyhedral structure, Co_3_S_4_, hydrogen evolution reaction, overall-water electrolysis

## Abstract

The advancement of overall water-splitting technologies relies on the development of earth-abundant electrocatalysts that efficiently produce H_2_ as a chemical fuel while offering high catalytic efficiency, structural robustness, and low-cost synthesis. Therefore, we aim to develop a cost-effective and durable non-noble electrocatalyst for overall water splitting. A straightforward hydrothermal approach was employed to fabricate freestanding polyhedral Co_3_O_4_ on a microporous Ni foam scaffold, followed by anion-exchange transformation in the presence of Na_2_S solution to yield its conductive sulfide analog. The engineered Co_3_S_4_ electrode delivers remarkable HER activity in 1.0 M KOH, requiring a low overpotential (<100 mV) to drive 10 mA cm^−2^, far outperforming its pristine oxide counterpart and even closely benchmarking with a commercial Pt/C catalyst. This exceptional performance is governed by the synergistic effects of enhanced electrical conductivity, abundant catalytic sites, and accelerated charge-transfer kinetics introduced through sulfur substitution. Furthermore, the optimized Co_3_S_4_ electrodes enable a bifunctional overall water-splitting device that achieves a cell voltage of >1.76 V at 100 mA cm^−2^ and maintains prolonged operational stability for over 100 hrs. of continuous operation. Post-stability analyses confirm insignificant phase preservation during testing, ensuring sustained activity throughout the electrolysis process. This study highlights the potential of anion-exchanged Co_3_S_4_ as a cost-effective and durable catalyst for high-performance HER and full-cell water-splitting applications.

## 1. Introduction

The accelerating depletion of fossil fuels and their associated environmental consequences, including global warming, greenhouse gas emissions, and air pollution, have raised serious concerns about our near-term energy needs, intensifying the demand for clean, renewable energy solutions [1,2,3,4,5,6,7]. Among various energy forms, hydrogen (H_2_) stands out as a carbon-neutral fuel with an exceptionally high energy density (142 MJ kg^−1^), offering significant promise for addressing the growing energy and climate challenges globally [8,9,10,11]. Electrochemical water splitting is regarded as one of the most sustainable and environmentally friendly technologies for achieving high-purity hydrogen production, as it avoids CO_2_ emissions that are inherent to the conventional steam methane reforming [12,13,14,15,16]. The hydrogen evolution reaction (HER) at the cathode is relatively straightforward compared to the oxygen evolution reaction (OER), yet its efficiency still relies heavily on the use of high-performance Platinum (Pt)-based catalysts, which still remains the benchmark catalyst for HER due to its near-optimal hydrogen adsorption energy and excellent catalytic activity in the electrolyte media [13,17,18]. However, its scarcity and high cost restrict its widespread utilization, necessitating the development of cost-effective, Earth-abundant alternatives that deliver comparable efficiency and long-term stability at higher current densities [19,20,21]. Further, owing to an industrial perspective, practical alkaline water electrolysis systems are typically required to deliver current densities above 500 mA cm^−2^ to achieve economically viable hydrogen production rates [22]. Therefore, beyond demonstrating electrolyzer cells’ activity at lower current rates, the development of robust electrocatalysts capable of sustaining high current densities with low overpotentials and long-term durability is crucial for industrial relevance.

Owing to their tunable redox chemistry, outstanding mechanical robustness, multiple valence states (Co^2+^ and Co^3+^), and structural stability, cobalt-based transition metal oxides (e.g., CoO_x_, Co_3_O_4_, CoO, and CoO_2,_ etc.) have garnered significant research interest as potential electrocatalysts for energy conversion processes [23,24,25,26,27,28,29]. Among them, Co_3_O_4_-based catalyst has been extensively investigated as an earth-abundant catalyst for electrochemical water electrolysis application [30,31,32,33,34,35,36]. However, the catalytic performance of Co_3_O_4_ is often hindered by its intrinsically low electronic conductivity and limited number of electrochemically accessible active sites in the bulk form, which restricts large-scale hydrogen production efficiency [37,38,39]. To overcome these drawbacks, tailoring the electronic structure and surface chemistry through sulfur-induced phase transformation has proven to be an effective strategy. This approach not only enhances the electronic conductivity (lowered resistivity ~10^−4^ Ω compared to its oxide counterpart) but also introduces abundant catalytically active sites in the active catalyst material, resulting in rapid electron transport, optimizes hydrogen adsorption–desorption kinetics, and lowers the energy barrier for water dissociation, thereby significantly boosting the HER activity [20,40]. This improvement is largely attributed to the electronic contribution of sulfur atoms, where the lone pair of electrons in the 3p orbital and the unoccupied 3d orbitals facilitate charge redistribution and result in the tuned electronic structure of the formed catalyst material [41,42,43].

In this study, we synthesized free-standing, binder-free, 3D polyhedral-like Co3O4 electrode films via a simple, facile hydrothermal method, followed by air-ambient annealing and subsequent anion exchange with Na_2_S to achieve the desired polyhedral-like Co_3_S_4_ electrode film. This transformation not only preserves the 3D polyhedral framework but also leverages the improved electronic conductivity and catalytic synergy introduced through the sulfur incorporation. The optimized Co_3_S_4_ catalyst electrode demonstrates significantly improved HER activity, as evidenced by a reduced overpotential of 91 mV at a current density of 10 mA cm^−2^ and a Tafel slope of 70 mV dec^−1^ compared to the pure Co_3_O_4_ (137 mV and 78 mV dec^−1^) catalyst. Further, the optimized polyhedral-like Co_3_S_4_ catalyst demonstrates excellent HER durability at varied applied current densities when examined under the alkaline KOH (1.0 M) condition. In addition, Co_3_S_4_ also served as a bifunctional electrode in an electrolyzer cell, demonstrating efficient full water-splitting capability, requiring full-cell voltages of 1.541 V, 1.758 V, and 2.11 V to drive current densities of 10, 100, and 500 mA cm^−2^, respectively. Moreover, the electrolyzer cell retains outstanding electrolysis stability, sustaining continuous hydrogen and oxygen generation over 100 hrs. of uninterrupted chronopotentiometric testing. These findings underscore the effectiveness of a versatile, scalable anion structural engineering strategy in developing cost-effective, robust, and intrinsically tuned catalysts with tunable kinetics, enabling the rational design of next-generation high-performance bifunctional catalysts for efficient and sustainable hydrogen production.

## 2. Materials and Methods

### 2.1. Materials

Acetone (CH_3_COCH_3_, ≥99.5%), ethanol (CH_3_CH_2_OH, ≥95%), cobalt(II) chloride hexahydrate (CoCl_2_·6H_2_O, ≥98%), potassium hydroxide (KOH, ≥85%), hydrochloric acid (HCl, 37%), thioacetamide (C_2_H_5_NS, ≥98%), and sodium citrate tribasic dihydrate (C_6_H_5_Na_3_O_7_·2H_2_O, ≥99%), were obtained from Sigma-Aldrich and used directly without further purification. Three-dimensional (3D) nickel foam (NF, 300 × 200 mm^2^, thickness = 1.6 mm, and cell size ≈ 450 μm) was purchased from Alantum (Seoul, Republic of Korea). Prior to use, the NF substrate was sequentially cleaned with acetone, diluted HCl, ethanol, and deionized water to remove surface impurities and enhance wettability.

### 2.2. Synthesis of Co_3_O_4_ and Co_3_S_4_ Electrodes

**Figure 1:**
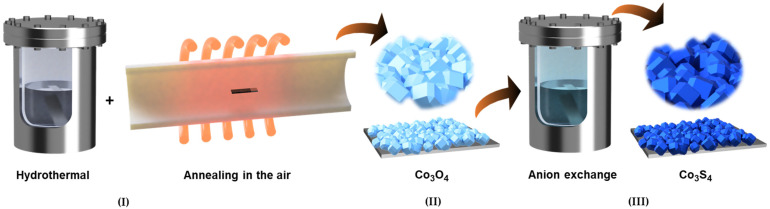
Schematic illustration of the synthesis process for three-dimensional polyhedral Co_3_O_4_ and its subsequent transformation into Co_3_S_4_ through anion-exchange. In the first step (**I**), Co_3_O_4_ polyhedral structures are obtained by hydrothermal growth of cobalt precursor, followed by controlled air annealing to induce crystallization and phase stabilization (**II**). In the following step (**III**), the Co_3_O_4_ template undergoes a sulfurization process in Na_2_S solution, where oxygen anions are gradually replaced by sulfide ions, leading to the formation of porous Co_3_S_4_ microstructures. Figure S1a shows the photograph image of Co_3_O_4_ and Co_3_S_4_ electrode films.

The fabrication of the desired Co_3_S_4_ electrode film was carried out using a two-step strategy: preparation of a Co_3_O_4_ template followed by an anion-exchange procedure (Figure 1). Initially, a hydrothermal reaction was employed to deposit Co_3_O_4_ onto a pre-cleaned NF substrate (deposited area = 1 × 1 cm^2^ and size = 5 × 1 cm^2^). In the typical procedure, 6 mmol of CoCl_2_·6H_2_O and 6 mmol C_6_H_5_Na_3_O_7_·2H_2_O were dissolved in 50 mL of deionized water under constant stirring. Subsequently, 18 mmol C_2_H_5_NS was introduced into the mixture, and the resulting solution was stirred for 30 minutes to ensure homogeneity. The NF substrate and precursor solution were transferred to a Teflon-lined stainless-steel autoclave, sealed, and maintained for 6 hrs. at 150 °C. After natural cooling, the as-prepared electrode film was collected and rinsed thoroughly with DI water and ethanol, then annealed in an air atmosphere at 400 °C for 2 h to form porous Co_3_O_4_. In the second step, the Co_3_O_4_ electrode film was immersed in 50 mL of 0.1 M Na_2_S solution and subjected to hydrothermal treatment for 10 h at 120 °C. This anion-exchange reaction transformed the oxide framework into microporous Co_3_S_4_ through the following reaction:

Co_3_O_4_ + 8 H_2_O + 4 S^2−^ ↔ Co_3_S_4_ + 4 H_2_O + 8 (OH)^−^, (1)

### 2.3. Material Characterization

X-ray photoelectron spectroscopy (XPS, ULVAC PHI 5000 VersaProbe, Chigasaki, Japan) was employed to determine the oxidation states and electronic environments of Co, O, and S. All binding energies were corrected with reference to the contaminant carbon C 1s peak at 283.89 eV. The morphology and elemental composition of the prepared electrode films were investigated using field-emission scanning electron microscopy (FESEM, JSM-6701F, JEOL, Tokyo, Japan) equipped with an energy-dispersive X-ray (EDX) detector. Crystallographic phases were analyzed by X-ray diffraction (XRD, Rigaku Smartlab, Akishima, Japan) operated at 40 kV and 30 mA with Cu Kα radiation (λ = 0.154056 nm) over a 2θ range of 20–80° at a scan rate of 2° min^−1^. Raman spectra were collected using a LabRam Aramis spectrometer (Horiba Jobin Yvon, Anyang, Republic of Korea) with a 514 nm Ar-ion laser excitation source to probe structural fingerprints and vibrational modes.

### 2.4. Catalytic HER and Bifunctional Activity

The hydrogen evolution reaction (HER) activity of the synthesized Co_3_O_4_ and Co_3_S_4_ electrode films was investigated using a VersaSTAT electrochemical workstation (Ametek Scientific Instruments, Berwyn, PA, USA) in a standard three-electrode setup with 1.0 M KOH as the electrolyte. The electrode films deposited on NF were employed as the working electrode, while a saturated calomel electrode (SCE, filled with KCl) and a graphite rod were used as the reference and counter electrode, respectively. Linear sweep voltammetry (LSV) was carried out in the potential window of 0.0 to −1.5 V (vs. SCE) at a scan rate of 1.0 mV s^−1^. The obtained potentials were converted into the reversible hydrogen electrode (RHE) scale using the following relation:

*E*_RHE_ = *E*_SCE°_ + (*pH* × 0.059) + *E*_SCE_, (2)

where *E*_SCE°_ is the standard potential (V) of SCE at room temperature. To minimize the effect of internal resistance, *JR* compensation was applied, and the HER overpotential (*η*) was estimated using the following equation:

*E*_RHE_ (*JR* compensated) = *η* = *E*_RHE_ − (*J* × *R*s), (3)

where *η* is the overpotential (V), *J* is the current density (mA cm^−2^), and *R*s is the solution resistance (Ω). The kinetic behavior was further analyzed by constructing Tafel plots from the linear portion of the LSV curves, which can be expressed as follows:

*η* = (*b* × log(*J*)) + *a*, (4)

where *b* is the Tafel slope (mV dec^−1^) and *a* is an arbitrary constant. To assess the electrochemically active surface area (*ECSA*), non-Faradaic cyclic voltammetry (CV) was performed over the potential range of 0.01 to −0.15 V (vs. SCE) at various scan rates. Electrochemical impedance spectroscopy (EIS) was conducted to probe the charge-transfer resistance. The EIS curves were measured at a negative bias potential over a frequency range of 0.01–10 kHz using an AC signal amplitude of 10 mV. Nonetheless, the chronopotentiometry was carried out to examine the long-term durability of the formed electrode films. Further, the bifunctional activity of the Co_3_S_4_ catalyst was examined in the same KOH condition, and the LSV, chronopotentiometric voltage step profile, and long-term stability were assessed to showcase the potential capability of the formed electrolyzer cell in the alkaline medium.

## 3. Results

### 3.1. Crystallographic and Bonding Properties of Co_3_O_4_ and Co_3_S_4_ Electrodes

**Figure 2:**
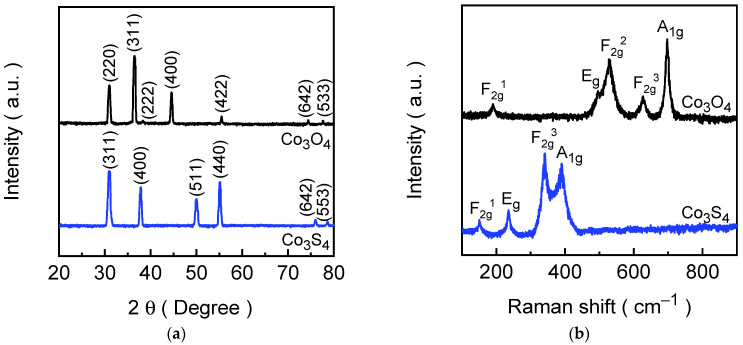
(**a**) XRD spectra; (**b**) Raman spectra of Co_3_O_4_ and Co_3_S_4_ electrode films.

The structural characterizations of the formed Co_3_O_4_ and Co_3_S_4_ electrode films are crucial for understanding their physical properties and potential applications in energy-related device applications. To investigate the crystallinity, phase purity, and structural transformations of the synthesized Co_3_O_4_ and Co_3_S_4_ films, XRD and Raman spectroscopy techniques were employed, which provide complementary insights into both the long-range crystalline order and short-range vibrational characteristics of the materials. Figure 2a presents the XRD patterns of the Co_3_O_4_ and Co_3_S_4_ electrode films, which showcase the well-defined diffraction peaks, indicating good crystallinity of the formed electrode films. For the Co_3_O_4_ electrode film (depicted in the black spectrum), the diffraction peaks observed at 31.01°, 36.52°, 38.27°, 44.55°, 55.49°, 74.42°, and 77.65° are indexed to the (220), (311), (222), (400), (422), (620), and (533) planes of Co_3_O_4_ phase with cubic crystal lattice, respectively [43]. These diffraction peaks aligned well with the standard JCPDS reference spectrum (card No. 76-1802), confirming the successful formation of a cubic spinel structure, which consists of Co^2+^ ions occupying tetrahedral sites and Co^3+^ ions in octahedral coordination with oxygen [44]. In contrast, the Co_3_S_4_ film (depicted in the blue spectrum) exhibits completely different diffraction peaks positioned at 31.04°, 37.84°, 50.05°, 55.21°, 76.00°, and 78.67°, which correspond to the (311), (400), (511), (440), (642), and (553) planes of cubic Co_3_S_4_ (JCPDS card No. 73-1703), respectively [45]. The XRD result confirms the successful transformation of Co_3_O_4_ into its sulfide-counter form through an ion-exchange process, while maintaining a cubic crystal system. The cubic symmetry of both Co_3_O_4_ and Co_3_S_4_ corresponds to the space group Fd$\bar{3}$m (227) is characterized by high structural stability and isotropic properties in the lattice. The lattice parameters and unit cell volumes provide additional insight into the structural differences between the oxide and sulfide phases. For the Co_3_O_4_ structure, the lattice constant (a) is calculated as 8.068 Å with a unit cell volume of 525.17 Å^3^. Whereas the lattice constant for Co_3_S_4_ is significantly increased to ~9.402 Å and possesses a unit cell volume of ~831.11 Å^3^. The expansion in lattice dimensions can be attributed to the larger ionic radius of sulfur compared to oxygen, which introduces longer Co–S bond lengths relative to Co–O bonds in the spinel framework [46]. Notably, the XRD spectra of both electrode films show no additional peaks corresponding to other cobalt-containing phases such as CoO, Co(OH)_2_, or elemental cobalt, confirming the phase purity of the synthesized materials.

Raman spectroscopy was further employed to examine the vibrational modes of the formed electrode films and to help understand insights into short-range order and local structural changes induced by the ion-exchange reaction. Figure 2b illustrates the Raman spectra of Co_3_O_4_ and Co_3_S_4_ electrode films. The Co_3_O_4_ electrode film exhibits five distinct Raman peaks at~192, 495, 527, 627, and 697 cm^−1^ corresponding to the F_2g_^1^, E_g_, F_2g_^2^, F_2g_^3^, and A_1g_ vibrational modes of the cubic spinel Co_3_O_4_ structure [47]. These vibrational modes arise from symmetric and asymmetric stretching and bending vibrations of the Co–O bonds in tetrahedral and octahedral sites, reflecting the integrity of the oxide lattice. After the conversion to Co_3_S_4_, a significant shift in Raman peak positions was observed, which is indicative of the modifications in bond strengths and coordination environments [48]. The Co_3_S_4_ electrode film displays Raman peaks at 151, 235, 342, and 380 cm^−1^ originating from F_2g_^1^, E_g_, F_2g_^3^, and A_1g_ vibrational modes of the cubic Co_3_S_4_ structure [49]. The downward shift in Raman peaks relative to Co_3_O_4_ is consistent with the replacement of oxygen by the heavier sulfur atom, which reduces vibrational frequencies due to increased atomic mass and altered bond stiffness [50]. Nonetheless, the absence of Co_3_O_4_-related vibrational modes in the Co_3_S_4_ spectrum attests to the effective replacement of oxygen with sulfur in the lattice structure.

### 3.2. Chemical Bonding States of Co_3_O_4_ and Co_3_S_4_ Electrodes

**Figure 3:**
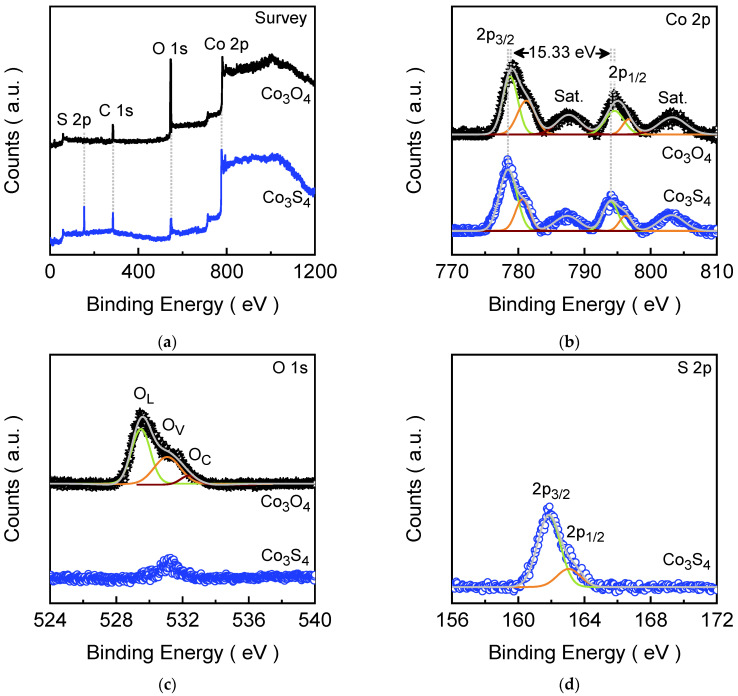
XPS spectra of Co_3_O_4_ and Co_3_S_4_ electrode films: (**a**) Survey spectra; (**b**) Narrow ranged Co 2p; (**c**) Narrow ranged O 1s; and (**d**) Narrow ranged S 2p spectra. All the narrow-range spectra were fitted using a Gaussian curve fitting model.

The XPS analysis plays a critical role in thoroughly characterizing the chemical states and surface composition of the prepared Co_3_O_4_-to-Co_3_S_4_ electrode films. For the as-prepared Co_3_O_4_ electrode film, an XPS survey spectrum (Figure 3a) confirms the presence of cobalt, oxygen, and carbon species. Figure 3a shows the core-level Co 2p spectrum, which exhibits four well-defined emission peaks. The intense characteristic peaks in the spectrum at lower binding energies, corresponding to the Co 2p_3/2_ and Co 2p_1/2_ states, were deconvoluted into two doublets. These peaks were situated at 778.79 and 781.25 eV (Co 2p_3/2_) and at 794.53 and 796.71 eV (Co 2p_1/2_), accompanied by satellite peaks at 787.62 and 803.23 eV. The observed energy separation of 15.74 eV between the Co 2p_1/2_ and Co 2p_3/2_ states is a characteristic of a mixed valence of cobalt, affirming the coexistence of Co^2+^ and Co^3+^ oxidation states in the spinel structure [51]. The O 1s core-level spectrum (Figure 3c) shows a broad emission peak, which was deconvoluted into three peaks corresponding to lattice oxygen (O_L_), oxygen vacancies (O_V_), and chemisorbed surface or dissociated oxygen species (O_C_) [52]. Notably, the Co 2p spectral characteristic of the Co_3_S_4_ electrode film (Figure 3b) strongly resembles that of the pristine Co_3_O_4_ electrode film; however, the emission peaks were slightly shifted toward lower binding energy, confirming that the core electronic structure around cobalt was largely preserved, aside from coordination changes (slightly improved Co^3+^/Co^2+^ states) during the phase transformation. Whereas the XPS emission spectrum of the Co_3_S_4_ electrode film obtained after anion-exchange process reveals an additional S 2p peak (Figure 3a) with a drastic reduction in the intensity of the O 1s peak, which nearly disappeared due to the oxygen replacement during the process. The characteristic S 2p emission signal was deconvoluted into the doublet, which emerged at 161.85 eV (S 2p_3/2_) and 163.04 eV (S 2p_1/2_). The spin energy separation of 1.19 eV of these degenerate states clearly indicated the presence of divalent sulfide ions (S^2−^) integrated within the new spinel lattice [53]. The minor residual of the O 1s peak was present, which likely formed due to air oxidation during the preparation of the sample. Thus, comprehensive XPS analysis validates not only the successful conversion of pure Co_3_O_4_ into Co_3_S_4_ but also clarifies the elemental, electronic, and surface transformations taking place throughout the process.

### 3.3. Morphological and Compositional Properties of Co_3_O_4_ and Co_3_S_4_ Electrodes

**Figure 4:**
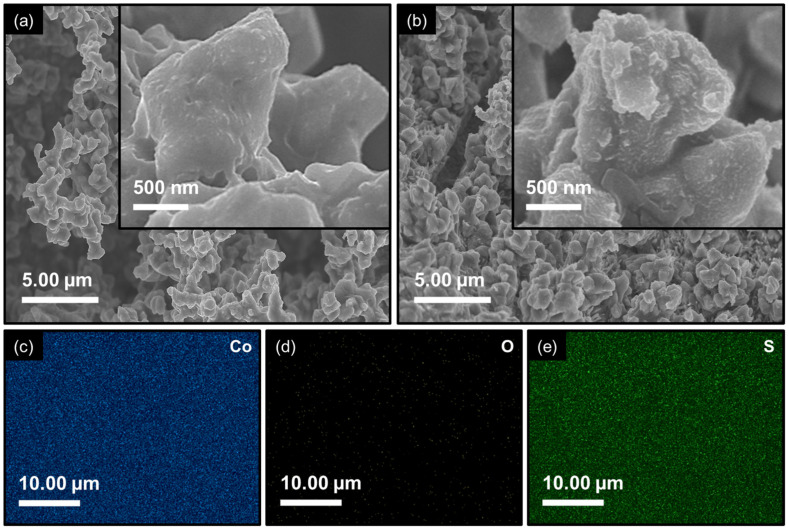
FESEM images recorded at low and high magnifications for (**a**) Co_3_O_4_ electrode film; (**b**) Co_3_S_4_ electrode film. FESEM-EDS elemental mapping images for the constituent (**c**) Co; (**d**) O; and (**e**) S elements.

The morphological and compositional properties of Co_3_O_4_ and Co_3_S_4_ electrode films were carefully examined using FESEM imaging and FESEM-EDS elemental mapping to understand the impact of an anion-exchange transformation on structural characteristics. Figure 4a reveals a well-defined three-dimensional polyhedral framework that is vertically oriented and randomly stacked on the 3D NF substrate. The uneven aggregation of polyhedrons creates noticeable voids among adjacent structures, which not only provide additional accessible surface area but also facilitate electrolyte diffusion pathways. The surface textures of these polyhedrons appear to be smooth. Whereas the remarkable structural evolution is observed upon phase transformation into Co_3_S_4_ (Figure 4b) through the anion-exchange process. The polyhedral structure displays significant variations in size and surface topography compared to the pristine Co_3_O_4_ electrode film, and the smooth surface facets are altered into distinctly rough embossed textures. These surface irregularities and micro-roughness arose from recrystallization during anion exchange and can act as additional active centers for electrochemical reactions. The elemental composition and its distribution were further confirmed by FESEM-EDS analysis (Figure 4c, Figures S1b,c and S2). The spectra and corresponding elemental maps highlight a uniform dispersion of Co and O in Co_3_O_4_, while the successful replacement of oxygen with sulfur in Co_3_S_4_ is clearly evident. The extracted atomic ratios (inset of Figure S1b,c) are in excellent agreement with the stoichiometric values of the respective phases, further verifying the complete structural conversion. This precise compositional tailoring through anion-exchange not only validates the synthetic strategy but also enlightens the observed morphological evolution and its anticipated contribution to electrochemical performance.

### 3.4. Electrochemical Properties of Co_3_O_4_ and Co_3_S_4_ Electrodes

**Figure 5:**
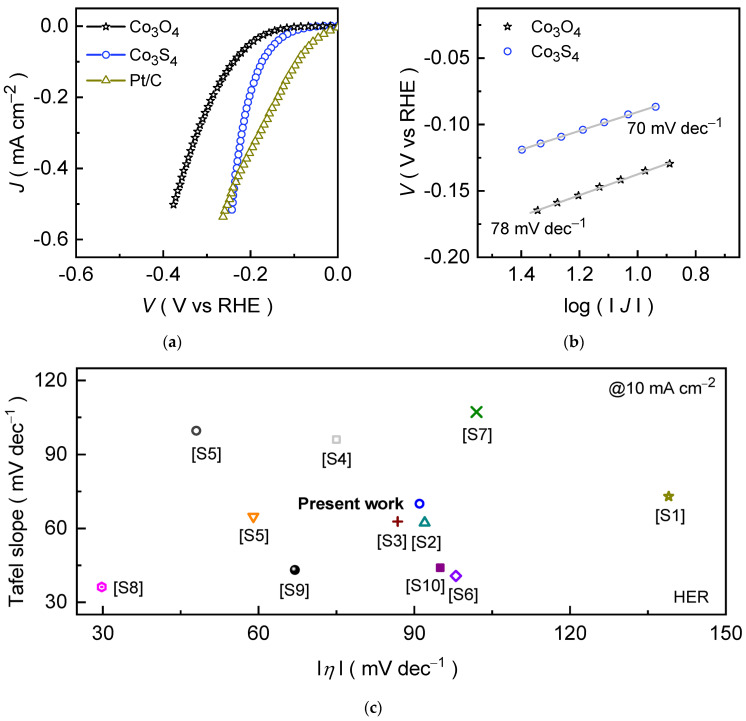
Electrocatalytic HER performance of Co_3_O_4_ and Co_3_S_4_ catalysts was examined in an alkaline 1.0 M KOH condition. (**a**) LSV curves; (**b**) Tafel slopes; and (**c**) Comparative HER performance of Co_3_S_4_ catalyst and reported metal sulfide-based catalysts at 10 mA cm^−2^ in 1.0 M KOH, with the additional details are summarized in Table S1.

The catalytic hydrogen evolution performance of Co_3_O_4_ and Co_3_S_4_ electrodes was characterized using the LSV curves recorded in the negative potential region. Figure 5a shows the *J* × *Rs* compensated polarization curves recorded in an alkaline 1.0 KOH medium for the prepared catalyst electrodes. The formed Co_3_S_4_ on NF substrate exhibits a markedly improved catalytic activity compared to the Co_3_O_4_ catalyst, requiring an overpotential of only 91 mV to deliver a current density of 10 mA cm^−2^. Whereas Co_3_O_4_ demands a higher overpotential of 137 mV to achieve the same current density. This enhancement becomes more pronounced at higher current densities. To drive the current densities of 20, 50, 100, 200, 300, 400, and 500 mA cm^−2^, the Co_3_O_4_ and Co_3_S_4_ catalysts require an overpotential of 111, 145, 173, 202, 219, 231, and 240 mV and 161, 201, 239, 288, 320, 349, and 375 mV, respectively. The remarkable HER activity of Co_3_S_4_ originates from its superior electrical conductivity (Figure S3a), efficient charge-transfer kinetics (Figure S3b), and enlarged electrochemically active surface area (Figure S4), as evidenced by reduced charge-transfer resistance in EIS and the enhanced double-layer capacitance values [54]. These findings indicate that substitution of “S” with “O” tailors the electronic structure, enhances electron mobility, and lowers energy barriers, thereby accelerating reaction kinetics and enabling highly efficient hydrogen evolution [55,56]. For benchmarking, the catalytic activities of bare NF (Figure S5a) and commercial Pt/C were also assessed under identical conditions. Undoubtedly, the Pt/C displays a low overpotential at a current density of 10 mA cm^−2^; however, the polyhedral Co_3_S_4_ reveals competitive HER activity at higher current densities, highlighting the potential capability of the formed Co_3_S_4_ catalyst. In contrast, NF substrate exhibits negligible HER activity across the entire potential range. Its primary role lies in providing a mechanically stable, high-surface-area scaffold for active catalyst deposition rather than contributing directly to the reaction. Further, the consistent HER activity observed across multiple Co_3_O_4_ and Co_3_S_4_ electrodes (Figure S6) further validates the reliability of the obtained catalytic properties. Taken together, these results demonstrate that the anion-exchange transformation of Co_3_O_4_ into Co_3_S_4_ significantly modifies the electronic structure and surface chemistry. As the sulfur substitution reduces the bandgap and increases the density of states near the Fermi level, enhancing charge transport and further introducing mixed-valence Co^3+^/Co^2+^ sites, which increases the number of active sites and exposed surface area, effectively boosts the HER performance in alkaline media by combining structural robustness with enhanced intrinsic activity [57].

To gain further insight into the catalytic mechanism, the Tafel slopes of Co_3_O_4_ and Co_3_S_4_ catalysts were analyzed. The HER in alkaline electrolytes proceeds through a two-step pathway involving water dissociation and hydrogen adsorption on the catalyst surface. Initially, the Volmer step occurs, where water molecules are reduced to generate adsorbed hydrogen species (MH_ads_) and hydroxide ions (OH^−^) [58]:

H_2_O + M + e^−^ → MH_ads_ + OH^−^ (theoretical Tafel slope = 120 mV dec^−1^), (5)

The subsequent hydrogen evolution can proceed either via the Heyrovsky step:

MH_ads_ + H_2_O + e^−^ → M + OH^−^ + H_2_ (theoretical Tafel slope = 40 mV dec^−1^), (6)

or by the Tafel step involving recombination of two adsorbed hydrogen atoms:

2 MH_ads_ → 2 M + H_2_ (theoretical Tafel slope = 30 mV dec^−1^), (7)

The experimentally obtained Tafel slope thus provides insight into the rate-determining step of the overall HER mechanism. Figure 5b shows the Tafel slopes of Co_3_O_4_ and Co_3_S_4_ catalysts obtained from the respective LSV curves (Figure 5a). The Co_3_S_4_ catalyst exhibits the comparatively lower Tafel slopes of 70 dec^−1^ compared to the pristine Co_3_O_4_ catalyst (78 dec^−1^), highlighting the superior kinetic performance of Co_3_S_4_ in an alkaline KOH medium and supported by the turnover frequency (TOF) analysis (Figure S3b). This improvement is primarily attributed to the sulfur substitution, which effectively modulates the electronic structure, enhances electrical conductivity, and facilitates rapid electron transport throughout the catalyst framework. In addition, strong synergistic interactions between sulfur and cobalt atoms (Co^3+^/Co^2+^ valence centers) enrich the density of accessible active sites, thereby accelerating proton discharge and simultaneously boosting hydrogen evolution. The fitted Tafel slope values for Co_3_S_4_ lie between the theoretical Volmer (120 mV dec^−1^) and Heyrovsky (40 mV dec^−1^) limits, indicating that the HER process predominantly proceeds through the Volmer–Heyrovsky mechanism [53].

To further evaluate the catalytic HER performance, the potential response of Co_3_O_4_ and Co_3_S_4_ catalysts was examined as a function of current density through chronopotentiometric measurements (Figure S5b). The applied cathodic current densities were systematically increased from −10 to −50 mA cm^−2^ with a step of 10 mA cm^−2^ and then doubled to 100 mA cm^−2^ and subsequently decreased up to 10 mA cm^−2^ in reverse order. Both catalysts exhibited a nearly static potential response during continuous operation, highlighting their robust endurance under sustained electrolysis conditions. Clearly, the Co_3_S_4_ catalyst consistently exhibits the lower potential values compared to the Co_3_O_4_ across the entire current range, highlighting its superior catalytic efficiency. The stable voltage plateaus at each step without noticeable drift confirm the strong interfacial contact between catalyst and substrate. Moreover, the nearly linear correlation between potential and current density suggests efficient charge transfer and minimal resistance losses, reinforcing the kinetic advantages of sulfur incorporation over the pristine Co_3_O_4_ catalyst. Notably, the low overpotential response of the Co_3_S_4_ catalyst is comparable to, or even better than, that of many recently reported transition-metal sulfide catalysts, which are summed up in Figure 5c. In addition to their catalytic activity, the long-term HER durability of Co_3_S_4_ catalyst was further assessed through chronopotentiometric stability measurements at an applied current density of −10 and −100 mA cm^−2^ for a prolonged duration of 100 hrs. (Figure S5c). Remarkably, the Co_3_S_4_ catalysts maintain a stable voltage profile throughout the test with negligible degradation in the potential response, reaffirming their superior structural integrity and charge transport properties during the extended HER operation. The post-stability measured FESEM and FESEM-EDS analyses (Figure S7) further confirm that the morphology and elemental composition (Co, S, and O) remain largely unchanged, supporting the observed electrochemical stability. The excellent stability can be ascribed to the intrinsic robustness of the Co_3_S_4_ phase and its firm interfacial bonding with the Ni foam substrate, which maintains efficient charge transport and active-site accessibility during prolonged operation.

### 3.5. Overall Water Splitting Properties

**Figure 6:**
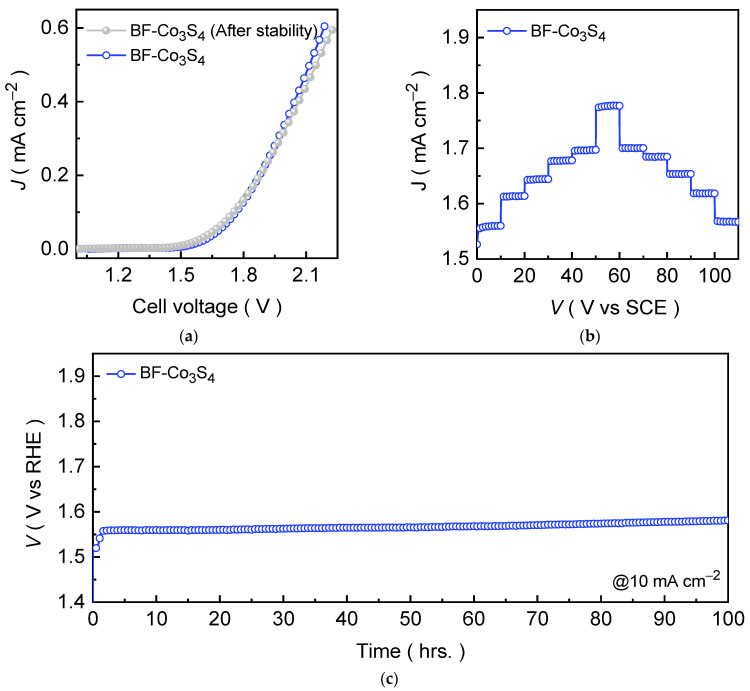
(**a**) LSV curves for the bifunctional Co_3_S_4_ (BF-Co_3_S_4_) electrolyzer cell recorded before and after the prolonged chronopotentiometric stability test; (**b**) voltage step profile for BF-Co_3_S_4_ electrolyzer at various current densities; and (**c**) chronopotentiometric stability profiles recorded at 10 mA cm^−2^ for 100 hrs. for the Co_3_S_4_ electrolyzers.

Owing to the excellent HER activity of the Co_3_S_4_ catalyst at diverse current densities, we fabricated an electrolyzer cell using a two-electrode setup. Figure 6a shows the LSV curve of an electrolyzer cell formed using the bifunctional Co_3_S_4_ (BF-Co_3_S_4_) catalyst measured at a scan rate of 1.0 mV s^−1^ in an alkaline 1.0 M KOH medium. The LSV curve demonstrates that the BF-Co_3_S_4_ electrolyzer cell requires only 1.541 V to achieve a current density of 10 mA cm^−2^. A continuous, vigorous gas evolution, clearly observed at the surfaces of both cathode and anode electrodes, confirms efficient hydrogen and oxygen generation with an excellent Faradaic efficiency of ~99% and 95%, respectively. At higher driving current density of 100 mA cm^−2^, an electrolyzer cell exhibits robust catalytic activity achieving cell voltage of 1.758 V. To further probe the relationship between potential and current density, voltage-step chronopotentiometric tests were also conducted by sequentially increasing the current density from 10 to 50 and 100 mA cm^−2^ with an increment of 10 and 50 mA cm^−2^, respectively, followed by a reverse step back to 10 mA cm^−2^ (Figure 6b). The voltage response remained nearly linear with minimal fluctuations at each applied current density, confirming the excellent electron/ion transport and conductivity throughout the catalyst network. The long-term stability was further verified by chronopotentiometric measurement at a current density of 10 mA cm^−2^. Figure 6c shows that the BF-Co_3_S_4_ electrolyzer sustained a steady voltage profile over prolonged electrolysis without noticeable degradation throughout the chronopotentiometric test, which is in good agreement with the post-stability measured LSV curve. These results collectively demonstrate that Co_3_S_4_ is a highly durable and efficient bifunctional electrocatalyst for overall water splitting application in an alkaline electrolyte medium.

## 4. Conclusions

In summary, the three-dimensional polyhedral Co_3_S_4_ electrocatalysts were successfully synthesized through an anion-exchange transformation of a Co_3_O_4_ template using the Na_2_S solution. The precursor Co_3_O_4_ structure was synthesized through a simple, cost-effective, and eco-friendly hydrothermal process followed by air annealing (400 °C for 2 h), and systematically evaluated for HER and bifunctional catalyst in an alkaline 1.0 KOH medium. Comparative electrochemical analyses revealed that the Co_3_S_4_ catalyst outperformed the pristine Co_3_S_4_ catalyst, exhibiting significantly lower overpotentials of 91 mV at a current density of 10 mA cm^−2^, a smaller Tafel slope (70 mV dec^−1^), and enhanced charge transfer properties. The improved catalytic HER performance of the Co_3_S_4_ catalyst is attributed to the higher conductivity, optimized electronic structure, and increased electrochemically active surface area induced by sulfur incorporation, which collectively accelerate the intrinsic reaction kinetics. Further, the chronopotentiometric voltage-step profiles confirmed a stable potential response over a wide current density range, while long-term stability measurements demonstrated excellent durability for 100 hrs. under sustained testing. The formed BF-Co_3_S_4_ two-electrode electrolyzer can operate over a wide current density range and achieves low cell voltages of 1.541 and 1.758 V at 10 and 100 mA cm^−2^, respectively. Moreover, the BF-Co_3_S_4_ electrolyzer cell demonstrates excellent chronopotentiometric endurance, with continuous and stable gas evolution over the long-term stability period (100 hrs.). These results underscore the potential of Co_3_S_4_-based electrodes as promising and scalable candidates for future hydrogen generation systems, particularly for alkaline electrochemical electrolyzers in renewable energy storage-conversion devices. In addition, sulfur-driven anion exchange provides an effective route to design advanced transition-metal chalcogenide catalysts with high activity, durability, and cost efficiency for sustainable, large-scale hydrogen production.

## Data Availability

The original contributions presented in this study are included in the article and Supplementary Materials. Further inquiries can be directed to the corresponding author.

## Supplementary Materials

The following supporting information can be downloaded at: https://www.mdpi.com/article/10.3390/ma18215025/s1. Figure S1: (a) Photograph of the deposited Co_3_O_4_ (left) and Co_3_S_4_ (right) electrode films. FESEM-EDS spectra of (b) Co_3_O_4_ and (c) Co_3_S_4_ electrode films. The inset tables summarize the corresponding elemental compositions in terms of atomic percentage ratios; Figure S2: FESEM-EDS elemental mapping of Co_3_O_4_ electrode film showcasing the uniform distribution of (a) Co and (b) O elements; Figure S3: (a) Nyquist impedance curves along with the tank circuit and (b) *TOF* curves for Co_3_O_4_ and Co_3_S_4_ catalysts; Figure S4: Scan rate dependent CV curves of the (a) Co_3_O_4_ and (b) Co_3_S_4_ catalyst films measured in non-Faradaic potential region at different scan rates. (c) “*J* versus *v*” plots obtained at 0.06 V (vs. SCE) from non-Faradaic CV curves to calculate the double-layer capacitance and *ECSA*; Figure S5: (a) LSV curve of NF substrate and Co_3_S_4_ catalyst film. (b) Voltage step profile of Co_3_O_4_ and Co_3_S_4_ catalysts measured at various current densities. (c) Chronopotentiometric stability of Co_3_S_4_ catalyst film at 10 and 100 mA cm^−2^; Figure S6: Reliability data of (a) Co_3_O_4_ and (b) Co_3_S_4_ catalysts measured for the series of samples in the same experimental conditions; Figure S7: FESEM images of Co_3_S_4_ catalyst film recorded (a) before stability and (b) after stability test. (c) FESEM-EDS spectra of the Co_3_S_4_ catalyst recorded before and after stability test; Table S1: Electrocatalytic HER performance of the optimized Co_3_S_4_ catalyst compared with other reported metal sulfide-based catalysts in alkaline 1.0 M KOH electrolyte at a current density of 10 mA cm^−2^. References [59,60,61,62,63,64,65,66,67,68] are cited in the Supplementary Materials.

## Abbreviations

The following abbreviations are used in this manuscript:

H_2_	Hydrogen
HER	Hydrogen evolution reaction
OER	Oxygen evolution reaction
Pt	Platinum
3D	Three-dimensional
NF	Nickel foam
XPS	X-ray photoelectron spectroscopy
FESEM	Field-emission scanning electron microscopy
EDS	Energy-dispersive X-ray
XRD	X-ray diffraction
SCE	Saturated calomel electrode
EIS	Electrochemical impedance spectroscopy
LSV	Linear sweep voltammetry
RHE	Reversible hydrogen electrode
*η*	Overpotential
*ECSA*	Electrochemically active surface area
CV	Cyclic voltammetry
Rct	Charge transfer resistance
TOF	Turnover frequency
