# Supplementary material for: Self-Supported Polyhedral-like Co3S4 Nanostructures Enabling Efficient High Current Hydrogen Evolution Reaction
Source: Materials (Basel). 2025 Nov 4;18(21):5025. doi: 10.3390/ma18215025 (PMC12608485; doi:10.3390/ma18215025)
Supplement: Supplementary file 1 [file materials-18-05025-s001.zip › materials-3933374-supplementary.pdf]

Supporting Information

Self-Supported Polyhedral-like Co_3S_4 Nanostructures Enabling Efficient High Current Hydrogen Evolution Reaction

Abu Talha Aqueel Ahmed [1], Sangeun Cho [1], Abu Saad Ansari [2], Yongcheol Jo [2] and Atanu Jana [1,*]

[1] Division of System Semiconductor, Dongguk University, Seoul 04620, Republic of Korea

[2] Nano Center Indonesia Research Institute, Puspiptek Street, South Tangerang, Banten 15314, Indonesia

[2] Department of Opto-mechatronics Engineering, Pusan National University, Busan 46241, South Korea

Corresponding Author: atanujanaic@gmail.com

Table S1. Electrocatalytic HER performance of the optimized Co_3S_4 catalyst compared with other reported metal sulfide-based catalysts in alkaline 1.0 M KOH electrolyte at a current density of 10 mA cm^{-2}.

No.	Catalyst film	Overpotential @10 (mA cm^{-2})	Tafel slope (mV dec^{-1})	Stability at *J* (*J* in mA cm^{-2})	Supporting Reference
1	Ni_2P/Ni_5P_4@CC	139 mV	73	84.9 % 100 hrs.@10	[S1]
2	Ni_2O-Co_2P@nanoframe	92 mV	62.3	30 hrs.@10	[S2]
3	NiPS-CoPS-Mo_xC	86.8 mV	62.8	12 hrs.@20	[S3]
4	MnB-CoGSS@CoCHNSs	75 mV	96	10 hrs.@10	[S4]
5	S-$Co(OH)_2$	48 mV	99.6	100 hrs.@100	[S5]
6	Se-$Co(OH)_2$	59 mV	64.8	100 hrs.@100	[S5]
7	Mn-Co-Fe-P	98 mV	40.68	72 hrs.@10	[S6]
8	Mn-N-Co_9S_8	102 mV	107.2	88.1 % 35 hrs.@100	[S7]
9	(c/o)-Co-Se-W	29.8 mV	36.2	160 hrs.@80	[S8]
10	Co_3Mn_3N/Co@PNC	67 mV	43.1	10 hrs.@10	[S9]
11	Fe-doped Co-LDH@MoS_2-Ni_3S_2	95 mV	44	10 hrs.@200	[S10]
**12**	**Polyhedral Co_3S_4**	**91 mV**	**70**	**100 hrs.@10 100 hrs.@100**	**Present work**

Supporting Figures

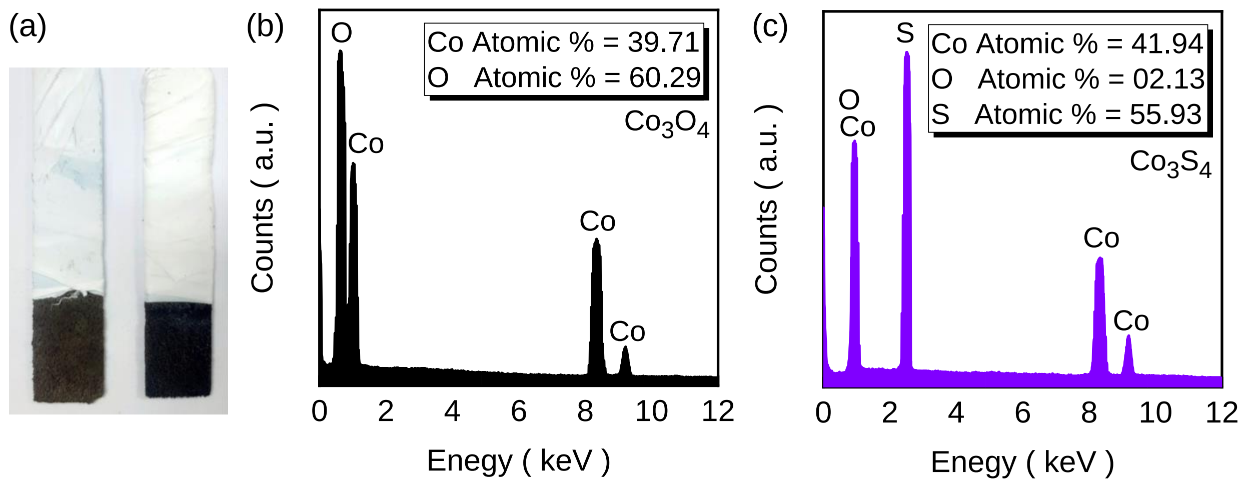

Figure S1. (**a**) Photograph of the deposited Co_3O_4 (left) and Co_3S_4 (right) electrode films. FESEM-EDS spectra of (**b**) Co_3O_4 and (**c**) Co_3S_4 electrode films. The inset tables summarize the corresponding elemental compositions in terms of atomic percentage ratios.

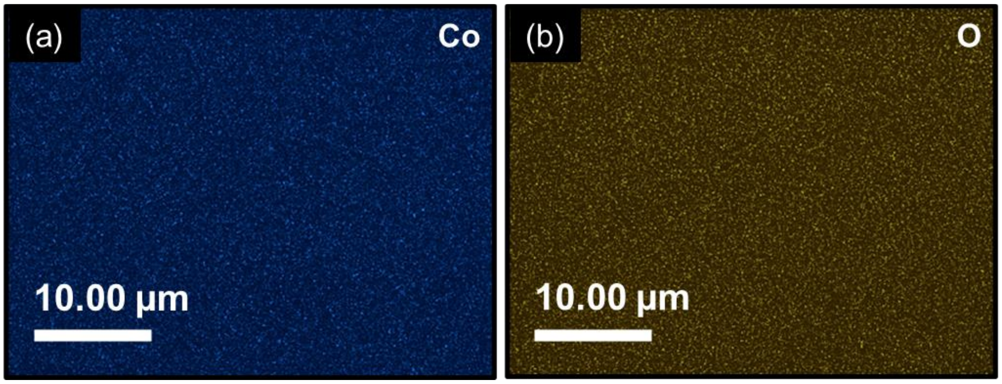

Figure S2. FESEM-EDS elemental mapping of Co_3O_4 electrode film showcasing the uniform distribution of (**a**) Co and (**b**) O elements.

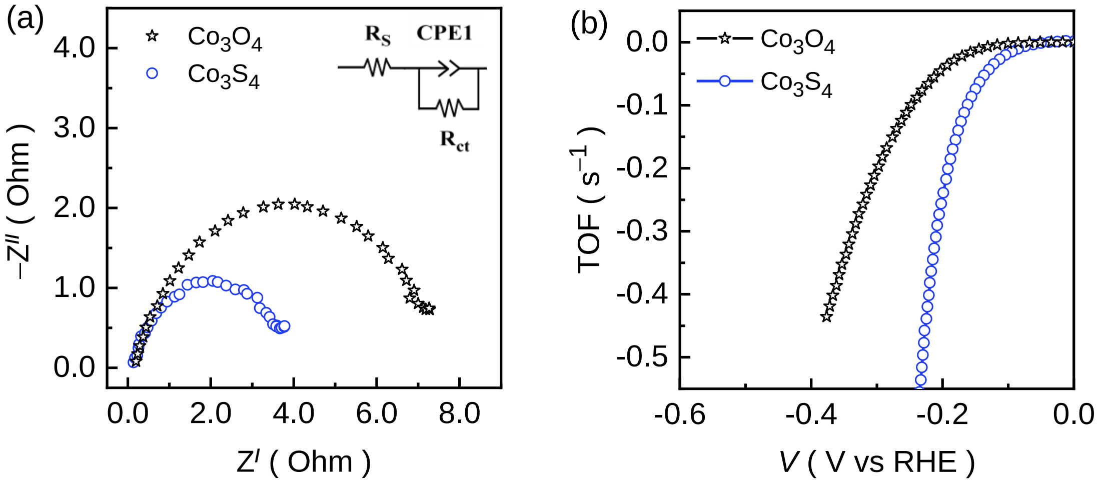

Figure S3. (**a**) Nyquist impedance curves along with the tank circuit and (**b**) *TOF* curves for Co_3O_4 and Co_3S_4 catalysts.

Electrochemical impedance spectroscopy (EIS) was performed to evaluate the charge-transfer characteristics of the Co_3O_4 and Co_3S_4 catalysts (Figure S3). The Nyquist plots of both samples exhibit a typical semicircular profile corresponding to the charge-transfer resistance (*Rct*) and the point at which the EIS curve intersects the X-axis, associated with the solution resistance (*Rs*). Notably, the Co_3S_4 catalyst displays a much smaller semicircle radius than Co_3O_4, demonstrating its lower charge-transfer resistance of 3.57 Ω for Co_3S_4 than Co_3O_4 (7.12 Ω). This substantial reduction in interfacial resistance highlights the beneficial impact of sulfur incorporation, which effectively tunes the electronic structure and enhances charge-carrier mobility, in good agreement with the TOF (Figure S3b) analysis. The improved conductivity of the catalyst collectively accelerates the charge transport at the electrode-electrolyte interface, thereby promoting rapid HER kinetics and improved overall electrocatalytic efficiency.

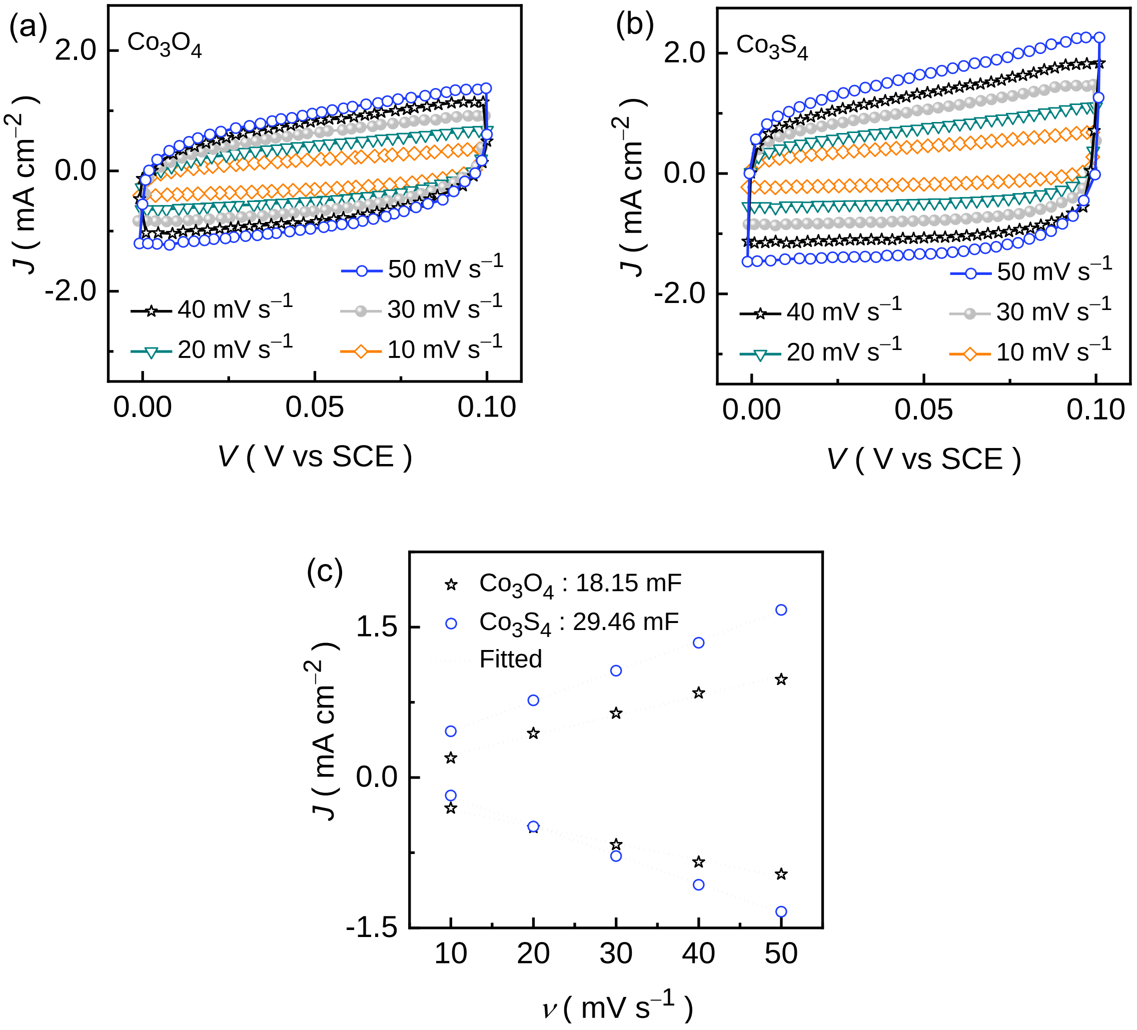

Figure S4. Scan rate-dependent CV curves of the (**a**) Co_3O_4 and (**b**) Co_3S_4 catalyst films measured in the non-Faradaic potential region at different scan rates. (**c**) "*J* versus *v*" plots obtained at 0.06 V (*vs*. SCE) from non-Faradaic CV curves to calculate the double-layer capacitance and *ECSA*.

The electrochemically active surface area (*ECSA*) was evaluated from the double-layer capacitance (Cdl) values obtained via CV curves recorded in the non-Faradaic region (Figure S4a,b). The linear dependence of current density (*J*) with scan rate (*v*) confirms capacitive behavior, from which the slopes yield the Cdl values. The Co_3S_4 catalyst exhibits a considerably higher Cdl of 29.46 mF (and therefore a larger ECSA) compared to Co_3O_4 (18.15 mF), indicating a substantially larger electrochemically accessible surface area. This enhancement can be attributed to the sulfur-induced phase transformation, which preserves the porous polyhedral framework while introducing abundant catalytic sites and improving intrinsic conductivity.

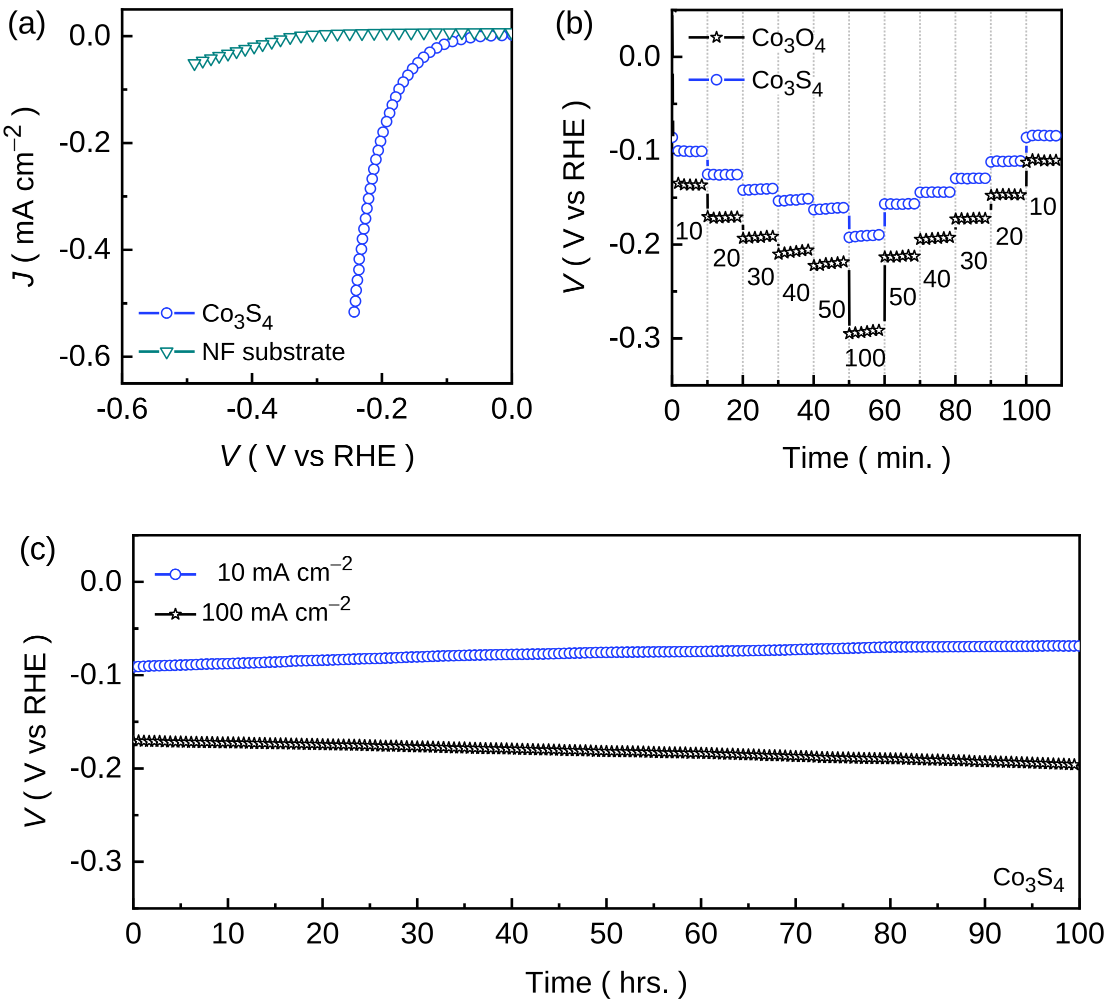

Figure S5. (**a**) Comparative LSV curve of NF substrate and Co_3S_4 catalyst film. (**b**) Voltage step profile of Co_3O_4 and Co_3S_4 catalysts measured at various current densities. (**c**) Chronopotentiometric stability of Co_3S_4 catalyst film at 10 and 100 mA cm^{-2}.

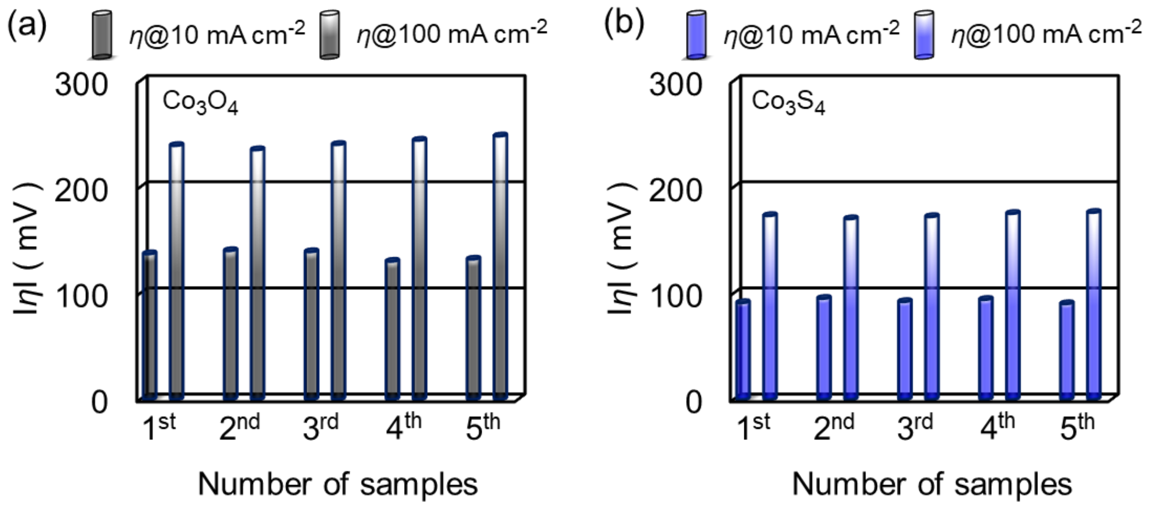

Figure S6. Reliability data of (**a**) Co_3O_4 and (**b**) Co_3S_4 catalysts measured for a series of samples in the same experimental conditions.

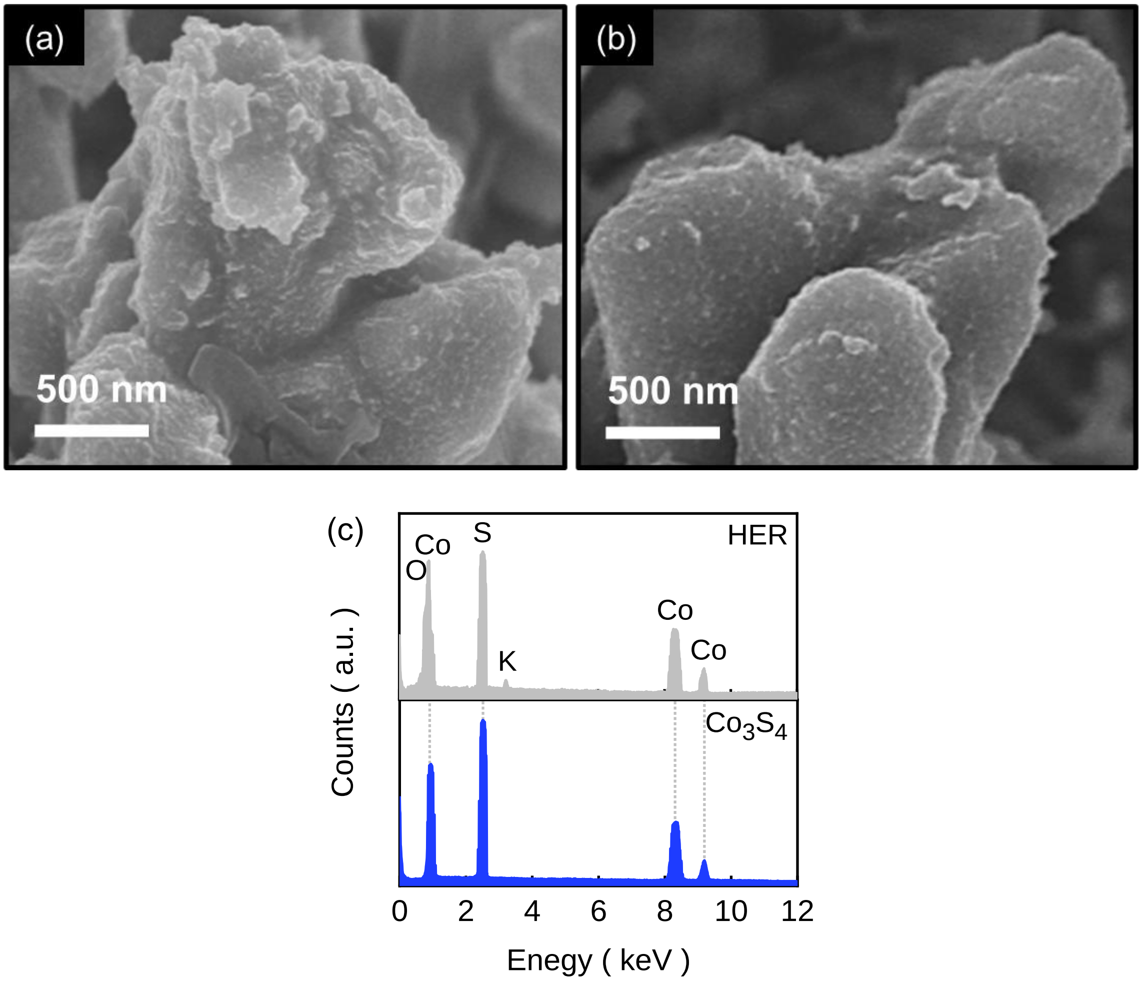

Figure S7. FESEM images of Co_3S_4 catalyst film recorded (**a**) before stability and (**b**) after stability test. (**c**) FESEM-EDS spectra of the Co_3S_4 catalyst recorded before and after stability test.

Supporting References

1. Huang, L.; Wei, X.; Yu, Y.; Sun, D.; Qu, Y.; Wen, J.; Yuan, X.; Su, Q.; Meng, F.; Du, G.; Wang, Y.; Xu, B.; Wang, K., In-situ construct Ni_2P/Ni_5P_4 heterostructured electrocatalyst through controllable Ni_2P phase transition for enhanced HER performance. *J. Mater. Sci. & Technol.* **2026,** *242*, 306-316.
2. Ji, L.; Wei, Y.; Wu, P.; Xu, M.; Wang, T.; Wang, S.; Liang, Q.; Meyer, T. J.; Chen, Z., Heterointerface Engineering of Ni_2P–Co_2P Nanoframes for Efficient Water Splitting. *Chem. Mater.* **2021,** *33* (23), 9165-9173.
3. Naseeb, M. A.; Murtaza, M.; Farooq, K.; Shah, W. A.; Waseem, A., Molybdenum carbide supported metal–organic framework-derived Ni, Co phosphosulphide heterostructures as efficient OER and HER catalysts. *Nanoscale Adv.* **2025,** *7* (17), 5300-5312.
4. Shamloofard, M.; Shahrokhian, S., ORR, OER, and HER activity promotion in hierarchical yolk–shell structures based on Co-glycerate@cobalt carbonate hydroxide by dual doping with manganese and boron. *Nanoscale* **2025,** *17* (34), 19695-19709.
5. Fan, C.; Song, X.; Tang, Y.; Zang, Z.; Ren, Y.; Li, L.; Yu, X.; Yang, X.; Lu, Z.; Zhang, X., S and Se-enhanced intrinsic activity of $Co(OH)_2$ for overall water splitting. *J. Alloys Compd.* **2025,** *1038*, 182648.
6. Wang, F.; Pei, Z.; Xu, Z.; Qin, T.; Ouyang, X.; Li, D.; Hou, Y.; Guo, X., Constructing Mn-Co-Fe Ternary Metal Phosphides Nanosheet Arrays as Bifunctional Electrocatalysts for Overall Water Splitting. *Adv. Sci.* **2025,** *12* (22), 2417521.
7. Xing, Y.; Li, D.; Li, L.; Tong, H.; Jiang, D.; Shi, W., Accelerating water dissociation kinetic in Co_9S_8 electrocatalyst by mn/N Co-doping toward efficient alkaline hydrogen evolution. *Int. J. Hydrogen Energy* **2021,** *46* (11), 7989-8001.
8. Zhang, J.; Cheng, C.; Xiao, L.; Han, C.; Zhao, X.; Yin, P.; Dong, C.; Liu, H.; Du, X.; Yang, J., Construction of Co–Se–W at Interfaces of Phase-Mixed Cobalt Selenide via Spontaneous Phase Transition for Platinum-Like Hydrogen Evolution Activity and Long-Term Durability in Alkaline and Acidic Media. *Adv. Mater.* **2024,** *36* (28), 2401880.
9. Lian, Y.; Lin, C.; Yu, T.; Qu, Y.; Yuan, C.; Guo, M., Regulative electronic structure of metallic Co_3Mo_3N/Co heterointerfaces in mesoporous carbon for decreased alkaline HER energy barriers. *Appl. Phys. Lett.* **2024,** *125* (10).
10. Phadikar, U.; Sanyal, G.; Das, S.; Kundu, A.; Kuila, C.; Murmu, N. C.; Chakraborty, B.; Kuila, T., Unique Multi-Hetero-Interface Engineering of Fe-Doped Co-LDH@MoS_2-Ni_3S_2 Nanoflower-Based

Electrocatalyst for Overall Water-Splitting: An Experimental and Theoretical Investigation. *ChemSusChem* **2024,** *17* (23), e202400821.
